# Mixed Cutaneous Infection Caused by Leishmania and Dermatophytes: A Rare Coincidence or Immunological Fact

**DOI:** 10.1155/2021/5526435

**Published:** 2021-03-08

**Authors:** Amresh Kumar Singh, Ankur Kumar, Jayesh Pandey, Vivek Gaur, Pratima Tripathi, Indra Prasad Adhikari

**Affiliations:** ^1^Department of Microbiology, Baba Raghav Das Medical College, Gorakhpur, Uttar Pradesh 273013, India; ^2^Department of Biochemistry, Baba Raghav Das Medical College, Gorakhpur, Uttar Pradesh 273013, India

## Abstract

Leishmaniasis was first described in 1824, in the Jessore district of Bengal (now Bangladesh) and more prevalent in Bihar, Uttar Pradesh, Jharkhand, and West Bengal. The disease is associated with depressed cellular immunity. Tinea is a fungal infection of the skin, which can become more extensively pathogenic particularly in patients with depressed cell-mediated immunity. Regulatory T cells and Th17 cells have been shown to be responsible for post-kala-azar dermal leishmaniasis (PKDL). We present a rare case of a 52-year-old house wife with a history of recurrent itching, depigmentation of the skin of extremities, and loss of appetite for 2-3 months followed by progressive spread of such lesion all over the body in an apparently healthy female. On examination, there were many hypopigmented scaly lesions mainly over the extensor aspect of the body. Skin lesions were characteristics of tinea infection with or without PKDL. A diagnosis of PKDL with tinea was made based on the history of kala-azar and on the skin slit smear for amastigote forms, *rK39* test, and KOH mount. Routine blood investigations showed negative serology for HIV and lower normal CD4+T counts. The patient was advised for treatment on systemic antifungal therapy with antihistaminics and later with miltefosine. We have highlighted that PKDL, although uncommon, is a distinct manifestation of VL. In our case study, we also tried to find the reason of coinfection; this was probably due to the depressed cellular immunity, skin abruptions, and acquired dermatophytic infection which is prevalent and associated with lower CD4+ T cell count.

## 1. Introduction

Visceral leishmaniasis (kala-azar) is a slowly progressing indigenous, neglected, and endemic disease caused by a protozoan parasite of the genus *Leishmania.* Leishmaniasis is a parasitic disorder transmitted by the bite of an infected female sandfly in developing countries, named *Phlebotomus* as per the National Vector Borne Disease Control Programme (NVBDCP) Govt of India and Lainson R. [1, 2]. The disease is transmitted by sandflies, which inoculate flagellated promastigotes into the skin of the host [3]. In human beings, leishmaniasis presents in four different clinical forms with different clinical manifestations: visceral leishmaniasis, or kala-azar; cutaneous leishmaniasis; mucocutaneous leishmaniasis; and diffuse cutaneous leishmaniasis [4].

Post‐kala‐azar dermal leishmaniasis (PKDL) is a late (usually posttreatment) complication of visceral leishmaniasis (VL) caused by *Leishmania donovani*. VL is one of the major neglected tropical diseases as per the World Health Organization (WHO), and it is especially prevalent among the poorest communities in endemic areas [5]. PKDL is a non‐life‐threatening cutaneous form characterized by a variety of skin manifestations, from hypopigmented macules to papular or nodular lesions. PKDL is generally linked to a previously occurring VL, and the lesions tend to appear 2-3 years after the completion of treatment in the Indian subcontinent [6]. The risk of developing PKDL seems to be associated with incomplete sodium stibogluconate (SSG) treatment [7]. However, PKDL has also been reported in patients treated with other antileishmanial drugs (i.e., miltefosine, amphotericin B, and paromomycin) and in individuals with no history of VL [6, 8, 9]. PKDL cases are relevant in the transmission of *L. donovani,* because they can act as reservoirs. However, except for cosmetic considerations, PKDL does not cause any physical limitation, and thus, patients do not tend to seek medical care for it. Active case detection is required to ensure adequate identification and treatment of patients [10].

PKDL is more prevalent in East Africa, where up to 50% of VL cases develop PKDL, whereas, on the Indian subcontinent, only 5%–18% of cases develop this cutaneous form [6]. However, the number of studies estimating the prevalence of PKDL in the Indian subcontinent is limited. A recent study showed that 2.4% of treated VL patients developed PKDL in Nepal but the exact prevalence is not known [7]. The PKDL incidence has been rising, and the estimated PKDL prevalence was 6.2/10,000 in Bangladesh [10, 11]. In India, to our knowledge, only one study was conducted in Uttar Pradesh and reported a PKDL prevalence of 48.2/10,000 in 1987 [12]. Therefore, the actual prevalence of confirmed PKDL cases was 1.8/10,000. Even if the incidence of PKDL seems to be decreasing in India according to surveillance data, there are no recent studies assessing the burden of PKDL in endemic areas [13].

Tinea corporis is a fungal infection that affects superficial layers of the skin caused by dermatophytes. It is defined based on the site of the lesions that may involve the trunk, neck, arms, legs, and groin. These include the scalp (tinea capitis), the face (tinea faciei), groin (tinea cruris), hands (tinea manuum), and feet (tinea pedis) [14]. Tinea corporis is common worldwide, and its prevalence is increasing day by day. Dermatophytes are the most prevalent causative agents of superficial fungal infections. There are many predisposing factors for rising prevalence: excessive heat, high relative humidity, and tight-fitted clothing [15]. Tinea can become more extensively pathogenic, particularly in patients with depressed cell-mediated immunity. T cell responses have been identified, such as regulatory T cells and Th17 cells that have been shown to be implicated in various forms of leishmaniasis as well as post-kala-azar dermal leishmaniasis (PKDL). The immunocompromised patients also have an increased prevalence of Majocchi granuloma, a type of tinea corporis folliculitis that invades the deep dermal layers [16].

Here, we are presenting a unique interesting case of mixed infection caused by *Leishmania* spp. and *Trichophyton rubrum* presenting with skin lesions.

## 2. Case Report

A 64-year-old female patient residing at Jagdishpur village, Deoria district, UP, India, a housewife, was referred from the District Government Hospital, Deoria, for diagnosis and management of “post-kala-azar dermal leishmaniasis.” The patient was attending our hospital in December 2020 complaining of recurrent itching, depigmentation of the skin of extremities, and loss of appetite for 2-3 months followed by a progressive spread of such lesion all over the body. Hypopigmented rashes were present all over the body for two months. The patient was apparently fine 2 years earlier when she started complaining of malaise, rashes, and loss of appetite on and off. She took local symptomatic treatment (paracetamol for malaise and multivitamins with unknown ointment) for about one month but did not get any relief and gradually started developing progressive itching lesions from the extremities to the anterior abdominal wall. After history taking, she has mentioned that she was diagnosed as a case of visceral leishmaniasis in 2017 based on history, epidemiology, visceromegaly, and *rk39* test. For that, she has completed treatment as per the guideline and she cured clinically.

**Figure 1:**
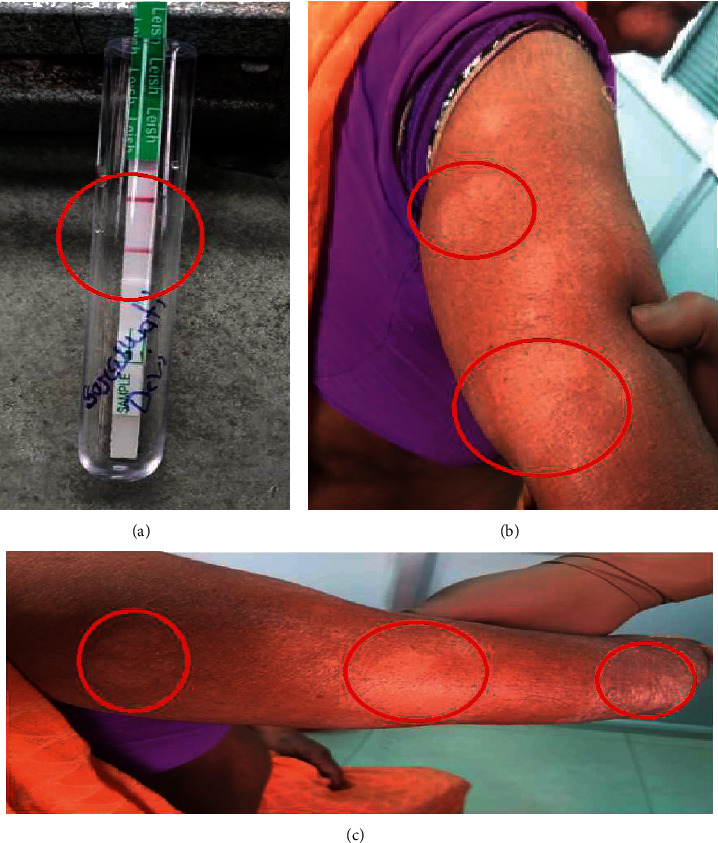
a) rK39 rapid card test (positive result). (b) and (c) Multiple hypopigmented lesions in the extensor aspect of upper limbs.

On examination, multiple hypopigmented, multiple circular, or ovoid in appearance lesions are present. These annular lesions demonstrate sharp marginations with raised erythematous scaly edges at a few places. In some of these lesions, the degree of inflammation was variable as seen in Figures 1(b) and 1(c). The lesions spread centrifugally, leaving a central clearing and mild residual scaling; this appears as a “ring” shape so, named as “ringworm.” Since this case was referred from the periphery for evaluation of PKDL, we evaluated as per this case the need and suspicion.

Thus, in lieu of suspicion for tinea and PKDL, we have planned to do a first skin scraping and then skin slit biopsy and serological test for leishmaniasis. The diagnosis of tinea corporis with PKDL was made based on a clinical thorough history and physical examination. However, testing was to be done to confirm the diagnosis.

### 2.1. Skin Scraping

**Figure 2:**
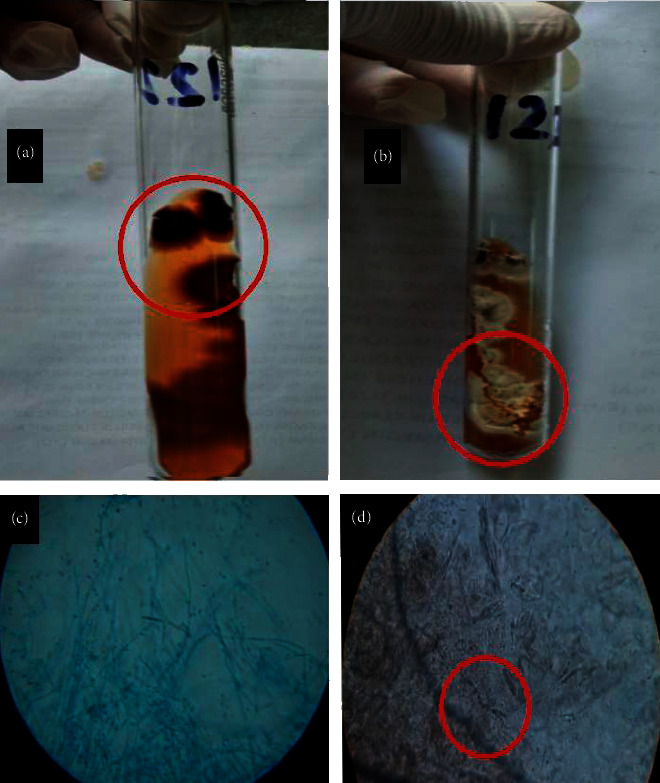
(a) Reverse side of fungal growth (*T. rubrum*) in SDA media. (b) Obverse side of fungal growth. (c) Lactophenol cotton blue (LCB) wet mount examination of fungal growth under high power microscopy (40X). (d) KOH mount examination of skin scarping under high power microscopy (40X).

A skin scraping was examined under a microscope with a potassium hydroxide (KOH) preparation which revealed hyaline, septate, and branching long narrow hyphae with occasional arthroconidia suggestive of dermatophytic infection as seen in Figures 2(b) and 2(c). This finding was supportive for the diagnosis of extensive dermatophytosis based on the extent of lesions. However, up to 15% of cases may yield false negatives when only using KOH preparations for diagnosis [17].

### 2.2. Fungal Culture

Therefore, another method for confirmation which is a fungal culture has been placed. Fungal cultures are important to diagnose genus and species but take time for definitive identification. The culture was done using skin scraping samples in Sabouraud dextrose agar (SDA). Cultures showed growth of the fungal pathogen in about five days. The colony was mostly flat to slightly raised, white to cream, suede-like to downy, with yellow-brown to wine-red reverse. The most common isolation medium used for fungal cultures is SDA (1% glucose, 4% mycological peptone agar, water). Identification was done by examining the morphology, pigmentations, and surface topography of the culture as a *Trichophyton rubrum* as seen in Figures 1(a) and 1(b) [18].

### 2.3. Lacto Phenol Cotton Blue-Stained Microscopy

**Figure 3:**
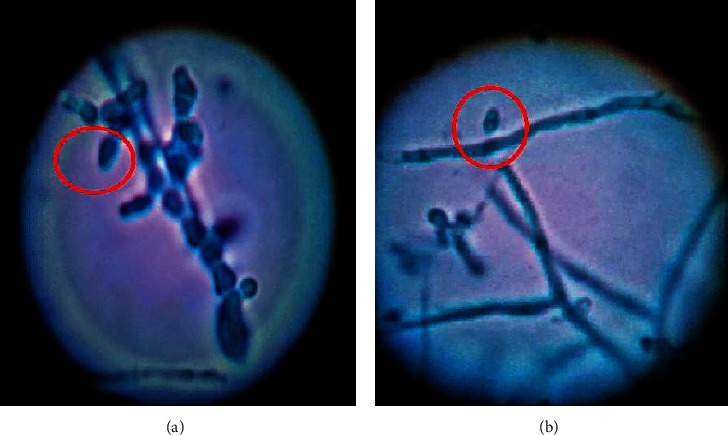
Lactophenol cotton blue mounted examination under 100X shows moderate numbers of slender clavate to pyriform microconidia suggestive of *T. rubrum*.

Most cultures show scanty to moderate numbers of slender clavate to pyriform microconidia under lactophenol cotton blue-stained wet smear made after teasing of fungal growth. Macroconidia are usually absent, but when present, they are smooth, thin-walled, multiseptate, slender, and cylindrical to cigar-shaped as seen in Figures 1(c), 3(a), and 3(b). The shape of microconidia varies from slender clavate to pyriform; the numbers of macroconidia range from none to scanty to many and may or may not have terminal projections.

### 2.4. Biochemical Test for Identification

Based on the battery of different morphological tests and biochemical tests like hydrolysis of urea (urease test) in broth and on urea agar slants and Petri plates incubated at 22°C, 28°C, and 37°C, *in vitro* hair perforation (blond child, sheep, and goat hair), pigment production on cornmeal dextrose agar (CMDA), and the genus and species level identification by biochemical tests like urease test as *T. rubrum* [19] were performed.

### 2.5. rK39 Rapid Immune-Chromatographic Test (ICT)

The *rK39* strip is a nitrocellulose strip impregnated with recombinant rK39 antigen and a commercially available test to diagnose *Leishmania* spp. infection (InBios International, Seattle, Washington, USA). One drop of serum from peripheral blood was applied at the base of nitrocellulose strips. After being air-dried, 2-3 drops of the test buffer (phosphate-buffered saline plus bovine serum albumin) were added, and the strip was placed upright for one minute. The appearance of a lower red band (control) indicates the proper functioning of the test, while the appearance of an upper red band indicates the presence of anti-rK39 IgG signifying a positive *rK39* test as seen in Figure 2(a) [20].

### 2.6. Skin Slit Smear

Collection of skin slit smear samples was performed using a sterile surgical blade, by a trained pathologist of our institute after taking proper consent. The important steps of this procedure were explained in detail. Local anesthesia (Xylocaine 1%) was given underneath the skin lesion at 3-4 places after sensitivity testing. Multiple imprint smears of the inner surface of the biopsy specimen were prepared immediately on three clean grease-free glass slides and fixed with methanol before staining.

### 2.7. Giemsa-Stained Microscopy

**Figure 4:**
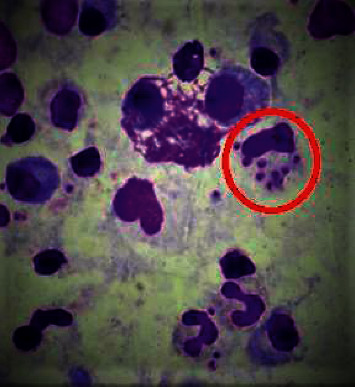
Giemsa-stained smear shows the typical presence of amastigote forms of *Leishmania* spp.

Giemsa-stained imprint smears were examined microscopically by two laboratory personnel for demonstration of Leishmania parasites (amastigote form), and the findings were confirmed by the pathologist. The histiocytes showed numerous intracytoplasmic amastigote forms of *Leishmania* spp. after Giemsa stain as shown in Figure 4 [21].

### 2.8. PCR Test for Leishmania spp

The skin specimen was cut from the filter paper with a disposable sterile scalpel and incubated in 250 *μ*l cell lysis buffer. DNA was extracted from the lysates with phenol-chloroform, and the pellets were air-dried. After being dissolved in 50 *μ*l Tris-EDTA buffer, the DNA was kept at 4°C until analyzed by PCR. The kinetoplast DNA (kDNA) PCR showed a positive result, correctly diagnosing as a confirmed positive sample. The kDNA PCR is considered to be the most sensitive method for diagnosing leishmaniasis.

### 2.9. Other Laboratory Findings

The routine laboratory investigations revealed anemia (HB: 8.1G/dl), mild thrombocytopenia (PLT: 0.9 lacs/cumm), and leukopenia (total leukocyte counts: 3300/cumm) with relative eosinophilia (E: 08%). In routine serology, HIV was negative, C-reactive protein was normal, CD4+T count was 670/cumm (lower side of normal range), and the ESR was peaked at 22 mm. The finding of chest X-ray was normal. Ultrasonography was performed to know the visceromegaly and it showed no evidence of visceromegaly.

### 2.10. Treatment Plan of PKDL with Tinea Infection

Based on laboratory findings, in this particular case, the first antifungal therapy with oral terbinafine with antihistaminics was started for two weeks, and after proper clinical cure, miltefosine will be given at the dose of 150 mg/day for 60 days [22]. Now, the patient has improved a lot, as far as hypopigmented lesions were improved, but currently, the patient is on miltefosine as per standard dose. We have called the patient, after the completion of antileishmanial therapy for microbiological assessment for a complete parasitic cure.

### 2.11. Differential Diagnosis

In this case, skin lesions were evaluated clinically, and the differential diagnosis may mimic the appearance of tinea corporis with or without PKDL. Cases that are refractory to unknown antifungal ointment and treatment should warrant further investigation to rule out other skin lesions. Other common differential diagnoses include PKDL, erythema annulare, centrifugum, nummular eczema, tinea versicolor, contact dermatitis, cutaneous candidiasis, subacute cutaneous lupus erythematosus, pityriasis rosea, atopic dermatitis, seborrheic dermatitis, and psoriasis.

## 3. Discussion

Tinea is a fungal infection of the skin, which can become more extensively pathogenic particularly in patients with depressed cell-mediated immunity. Visceral leishmaniasis is also associated with depressed cellular immunity [23]. This may be the reason for the mixed infection caused by *T. rubrum* and *Leishmania* spp. Immune responses in leishmaniasis are often described in terms of (T helper) Th1 and Th2 cellular immune responses [24]. In addition, other T cell responses have been identified, such as regulatory T cells and Th17 cells that have been shown to be implicated in various forms of leishmaniasis as reported by Zijlstra [25]. After treatment with antileishmanial drugs, the immune response changes to a predominantly Th1 response characterized by the presence of IL-12 production and INF-*γ* production by T cells. Macrophage activation with the subsequent killing of parasites is a key factor in disease control [26]. PKDL is characterized by an intermediate position between a Th1 and Th2 response, and this transition stage may govern the typical clinical features. As a result of antileishmanial treatment of leishmaniasis, the PKDL patient was no longer systemically ill and has no fever, and the liver and spleen are normal, as seen in this case. Only the amastigote forms of parasites persist that may have been there since VL [27].

The immune response to dermatophytes ranges from a nonspecific host reaction to a humoral and cell-mediated immune response. The currently accepted fact is that a cell-mediated immune response is responsible for dermatophytosis control. On the other hand, some individuals develop a chronic or recurrent infection mediated by the downregulation of cell-mediated immune response. Understanding the nature and function of the immune response to dermatophytes is a unique challenge that might lead to novel approaches in the research of treatment and immunological prophylaxis for dermatophytosis as shown by Almeida [28].

Although, *Trichophyton rubrum* was found in 20% of cases of dermatophytic infection in this geographical region in a previous study conducted for etiology of dermatophytes [29]. The *Trichophyton* spp. was also subjected to a urease test for the differential identification of *T. mentagrophytes* (showing a positive result) and *T. rubrum* (having a negative result) as in this case [19, 29]. *Trichophyton rubrum* is an anthropophilic fungus that has become the most widely distributed dermatophyte. Therefore, coinfection must be seriously considered, especially in patients who are supposed to be immunocompromised. Any hypopigmented patches and cutaneous papular polymorphic skin lesions must be investigated by the clinician in this endemic zone of leishmaniasis and some degree of dermatophytes; hence, for effective control of VL and to interrupt the transmission of kala-azar infection, its reservoir host PKDL needs to be detected early and treated adequately in the eastern part of Uttar Pradesh which is closely connected with the endemic zone of the Bihar state of India [30].

While reporting this case, we have highlighted the fact that PKDL, although uncommon, is a distinct manifestation of VL. Accurate early identification and management of these cases are crucial in eliminating a potential source of disease in the future. In our case study, we also tried to find the source of coinfection; this was probably due to the patient primarily suffering from PKDL and skin abruption, an acquired dermatophytic infection which was prevalent in this geographical region, and other factors (mainly immunological) as in this case because of lower CD4+T cell count also contribute to acquiring such mixed cutaneous infection. This may be because of the fact that these cases belong to rural areas and the majority of the patients consulted first with traditional healers or quack practitioners before being referred to a higher medical health facility. The patient was living in a house made up of stone and mud in an endemic area of eastern UP. Further, they also had a cattle shed in their yard all with conditions favorable for sand fly breeding as reported previously [31]. Even though kala-azar is endemic in the eastern UP and adjoining north-western part of Bihar, this patient was remained undiagnosed for a considerable period of time. Moreover, other risk factors which include lower socioeconomic status, lack of awareness about vector-borne or zoophilic infections, and mismanagement are the major causes that contribute to the high prevalence of these infections [32].

Limitations of this case report are the following. All immunological parameters were not analyzed to know the level of depressed cellular immunity except CD4+T cell count. Second, we have not analyzed the follow-up response of this case as this particular patient was first advised of oral antifungal therapy with antihistaminics and later planned for treatment with an antileishmanial drug.

Moving forward, this report will be useful to physicians as well as health policymakers on how to identify and diagnose such coinfection of dermatophyte with or without a history of VL. The technique used to identify and diagnose the suspected cases was easy to perform at any basic laboratory or clinical setup for early diagnosis and it also helps the clinician for the management of mixed PKDL and dermatophytic infections. As a final note, this case was rare and unique while considering cutaneous infection etiology could be mixed with PKDL and dermatophytes in India; therefore, clinicians as well as microbiologists should consider this approach during diagnosis and treatment management of the unusual cases of PKDL.

## Data Availability

The data used to support the findings of this study are available from the corresponding author upon request.
